# Nodular Esophageal Xanthoma: A Case Report and Review of the Literature

**DOI:** 10.1155/2017/1503967

**Published:** 2017-04-10

**Authors:** Ahmed Dirweesh, Muhammad Khan, Sumera Bukhari, Cheryl Rimmer, Robert Shmuts

**Affiliations:** ^1^Department of Internal Medicine, Seton Hall University, Hackensack Meridian School of Medicine, Saint Francis Medical Center, Trenton, NJ, USA; ^2^Department of Pathology, Our Lady of Lourdes Hospital, Willingboro, NJ, USA; ^3^Department of Gastroenterology, Our Lady of Lourdes Hospital, Willingboro, NJ, USA

## Abstract

Xanthomas are localized nonneoplastic lesions within tissues that may manifest as papules, plaques, or nodules. These lesions can be found anywhere along the gastrointestinal tract, commonly in the stomach and colon, and rarely in the small intestine and esophagus. Esophagogastroduodenoscopy (EGD) with biopsy is the gold standard tool for diagnosis. Here, we report a rare case of a lower solitary nodular esophageal xanthoma in an elderly black female. Correspondingly, all cases of esophageal xanthomas reported in the English medical literature were reviewed and presented with the reported case.

## 1. Introduction

Xanthoma (Greek word xanthos meaning “yellow”) is an uncommon nonneoplastic lesion resulting from the accumulation of foamy lipid‐laden histiocytic cells that can appear anywhere in the body. Most commonly, xanthomas are seen in the skin and tendons, while visceral xanthomas are uncommon. However, their histopathological features are identical regardless of location [1]. The gastrointestinal xanthomas were first described in 1887 as “lipid-laden macrophages in gastric mucosa.” The importance and etiology of gastrointestinal xanthomas remain largely unclear [2]. Understanding the endoscopic and pathologic features of these lesions is crucial for their detection and as a differential diagnosis of other pathologies as that may help physicians to appropriately manage these lesions.

## 2. Case Presentation

**Figure 1:**
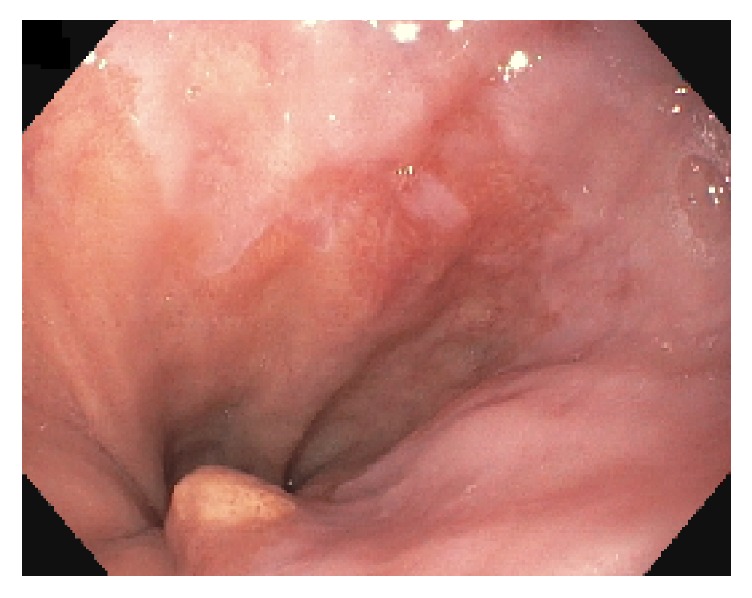
EGD showing a polyp (nodule) at the *z*-line.

A 60-year-old African American female presented with complaints of early satiety. She denied difficulty or pain with swallowing, reflux, vomiting, or regurgitation. She had a past medical history of diabetes mellitus, hypertension, hyperlipidemia, and tubular colonic adenoma. She also had an upper GI series showing questionable delayed esophageal drainage and thickening of esophageal folds concerning possible underlying delayed emptying and possible gastroparesis. She denied tobacco and drug use and consumes alcohol on rare occasions. On examination, vital signs were within normal ranges, and BMI was 36. The rest of the physical examination was normal. Based on her presenting complaints, she underwent an esophagogastroduodenoscopy (EGD) and a gastric emptying scan. EGD was positive for a polyp/nodule at the *z*-line together with a 1 cm hiatus hernia and gastritis (Figure 1).

**Figure 2:**
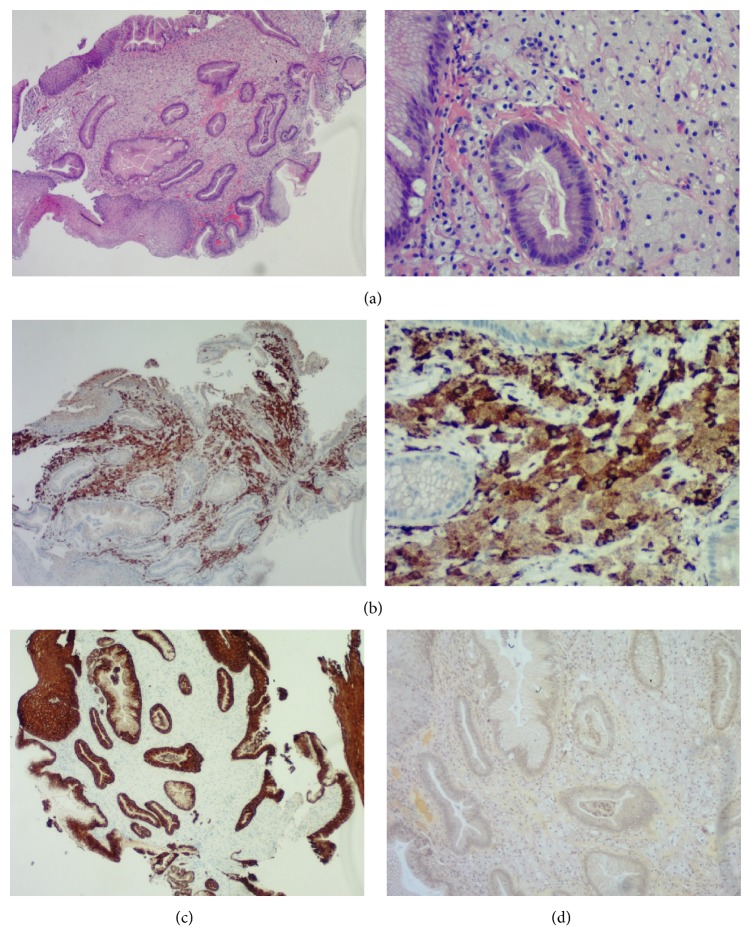
(a) Squamous and gastric mucosa with foamy histiocytes in the lamina propria (low and high power). (b) CD68 stain (low and high power) staining histiocytes brown in lamina propria. (c) A negative AE1/AE3 stain ruling out an epithelial lesion (carcinoma) as shown by staining the epithelium but the histiocytes in the lamina propria were negative. (d) A negative mucicarmine special stain ruling out signet ring cell carcinoma.

Biopsy of the esophageal nodule showed numerous foamy histiocytes in the lamina propria consistent with xanthoma (Figures 2(a), 2(b), and 2(c)).

## 3. Discussion

Gastrointestinal xanthomas are rare, smooth, yellowish tumor-like benign lesions. They can be incidentally discovered in the upper gastrointestinal tract during endoscopy [3]. The incidence of upper gastrointestinal xanthomas was reported as 0.23% [2]. Though the incidence in the upper gastrointestinal tract may vary among endoscopy series, the most frequent location is the stomach [3], followed by the duodenum and esophagus. One study showed the most common location of ectopic xanthoma in the gastrointestinal tract was the stomach (76%), followed by the esophagus (12%) and duodenum (12%) [2]. Xanthomas are considered as the sign of aging by few authors as the incidence of xanthomas increases with age. The incidence of 53.3% is in the age group of 40–60 years, although it can be seen in people of all ages [2].

Xanthomas are usually asymptomatic and can go undetected if the patient has no associated GI lesions. Their presence may be a manifestation of a metabolic disturbance, such as hyperlipidemia, or can be associated with other conditions such as previous radiotherapy, chemotherapy, and infection [cytomegalovirus (CMV) colitis and disseminated mycobacterium avium intracellulare (MAI)] in immunosuppressed patients (AIDS) [17]. However, it usually represents an isolated phenomenon. The correlation between lipid metabolism disorders and gastrointestinal tract xanthoma is not obvious [18, 19]. Many clinicians believe that yellowish plaque in the gastric mucosa is a benign lesion, and it has no clinical significance. But it can be mistakenly missed for a malignant underlying condition until unless proven by negative biopsy. It is important to distinguish xanthoma on endoscopy from other yellowish lesions such as carcinoid tumor, granular cell tumors, and ectopic sebaceous glands (ESGs).

It has been proposed that a proportion of gastric xanthomas may be provoked by* H. pylori* infection. Arima et al. reported that the prevalence of* H. pylori* infection was significantly higher in patients with gastric xanthomas compared to those without the disease [8, 9].

During endoscopy, xanthomas are shown to be small (1-2 mm) single or multiple yellow, orange, or white well-demarcated sessile macules with irregular outlines that rarely exceed 5 mm [1] though the larger lesions can be seen which may be nodular and elevated [6]. Microscopically, they are composed of compactly aggregated nests of large periodic acid-Schiff- (PAS-) negative round cells with small nuclei and foamy cytoplasm [1]. Most cells are histiocytes, although few other cells like plasma cells, smooth muscle cells, and Schwann cells may add to the entire picture [2].

The importance and etiology of gastrointestinal xanthomas remain largely unclear [2]. Few theories have been proposed by authors, explaining the possible trigger or etiology behind the pathogenesis of xanthomas. Mucosal injury has been presumed to contribute significantly to their pathogenesis as it yields lipid-containing debris, ultimately phagocytized by histiocytes forming foam cells [1]. This hypothesis would clarify why gastric xanthomas seem, by all accounts, to be more successive than esophageal xanthomas, as traumatism and irritation might be better endured by esophageal squamous epithelium than by gastric columnar epithelium [10]. Biliary reflux could be a fundamental etiological part [6].

The clinical significance of xanthomas arises due to the resemblance of features on endoscopy with other benign or malignant lesions. Understanding the endoscopic and pathologic features of xanthomas and other lesions is crucial for their detection and differential diagnosis as that may help physicians to appropriately manage these lesions. Differential diagnosis includes poorly differentiated carcinoma, storage diseases, infections (Whipple disease, mycobacterium, and AIDS), macroglobulinemia, and muciphages. The clinical picture together with the past medical history, symptoms of storage diseases, AIDS, and/or macroglobulinemia, is essential. In addition, special stains such as Gram, Ziehl-Neelsen, Gomori methenamine silver, PAS, and PAS-Diastase and immunohistochemistry for cytokeratin AE1–AE3 can be helpful [17].

Benign esophageal lesions have a broad range of clinical and pathologic features. The prevalence of benign esophageal tumors is under 0.5%, but they signify 20% of esophageal neoplasms on autopsy. With the widespread use of endoscopy, radiologic imaging, and increased awareness of the disease, these lesions could be detected more often [15]. Tsai et al. studied 2,997 patients and observed the frequency of benign epithelial and subepithelial lesions occurring in esophagus. In epithelial lesions, the frequency of occurrence was in the following order: glycogenic acanthosis, heterotopic gastric mucosa, squamous papilloma, hyperplastic polyp, ectopic sebaceous gland, and xanthoma. In subepithelial lesions, the order was as follows: hemangioma, leiomyoma, dysphagia aortica, and granular cell tumor [15].

Esophageal xanthomas, like all upper gastrointestinal tract xanthomas, are asymptomatic, the patients being usually investigated for other conditions. The first reported case occurred in the upper esophagus and was defined as “lipid islands” in 1984 by Remmele and Engelsing [4]. To the best of our knowledge, from 1984 to present, only 21 cases (including the presented one) have been reported (Table 1).

Data from Table 1 reflect the fact that most of these lesions were solitary, were less than 1 cm in size, and could be identified in all the three parts of the esophagus. The largest one of all was reported by Salamanca et al. as a verruciform growth in the upper esophagus in an elderly patient with a history of radiation exposure [12].

Esophageal xanthomas have to be grossly distinguished from ectopic sebaceous glands and small subepithelial tumors such as carcinoid and granular cell tumor because most of the reported esophageal xanthomas are yellowish or white mucosal elevated lesions. In terms of microscopic findings, signet ring cell carcinoma, which contains round cells with abundant cytoplasm, should be distinguished. While signet ring cell carcinoma has an eccentrically located nucleus because of the intracellularly abundant mucin, xanthoma has a centrally located and small nucleus. Accumulation of foamy histiocytes of xanthoma could be a clue for the differential diagnosis. In questionable cases, immunohistochemical stains for CD68 can be performed, which indicates a histiocytic origin, another characteristic finding of xanthoma [1]. Besides, esophageal malignancy and ectopic sebaceous glands do not commonly stain with Lugol's solution; consequently, endoscopists should be aware of these lesions for the differential diagnosis [20, 21]. The differential diagnosis becomes clearly inconclusive when biopsies are taken too superficially, permitting just assessment of the epithelium. It is in this way imperative that in any event some lamina propria is available in the biopsy example.

## 4. Conclusion

In spite of the fact that the etiology and clinical significance of gastrointestinal xanthomas are still obscure, determination of these lesions is imperative since they might coexist with malignant lesions. Having endoscopic resection of all granular cell tumors and squamous papillomas in light of the fact that, while uncommon, these lesions have malignant potential is suggestive.

## Figures and Tables

**Table 1:** Characteristics of reported esophageal xanthomas.

Number	Author(s)	Sex/age (years)	Location	Number of lesions	Size (mm)	Macroscopic findings	Associated medical history
(1)	Remmele and Engelsing [4]	Male/54	Upper	1	10	Yellow spot	Gastrectomy
(2)	Stolte and Seifert [5]	Male/45	Middle	3	<1	Yellow flat elevations	Hyperlipidemia, diabetes mellitus
(3)	Vimala et al. [6]	Female/37	Lower	Multiple	2–5	Yellowish nodular	Gastric xanthoma
(4)	Hirokawa et al. [1]	Female/52	Lower	1	2	Yellowish granular	Duodenal ulcer
(5)	Hirokawa et al. [1]	Male/67	Lower	1	2	Yellow spots	Hepatocellular carcinoma, hypertension
(6)	Herrera-Goepfert et al. [7]	Male/61	Middle	1	5	Verruciform	Non-Hodgkin lymphoma of the testis
(7)	Gencosmanoglu et al. [2]	Not specified	Not specified	Multiple	<5	Yellow-white colored plaques	Not specified
(8)	Gencosmanoglu et al. [2]	Not specified	Not specified	1	<5	Yellow-white colored plaques	Not specified
(9)	Gencosmanoglu et al. [2]	Male/49	Lower	1	3	Yellowish elevated granular lesion	Atrophic gastritis
(10)	Arima [8]	Male/74	Middle	1	4	Yellowish white patch	Not specified
(11)	Arima [8]	Male/74	Upper	1	2	Whitish protruding lesion	Not specified
(12)	Licci et al. [9]	Male/49	Upper	1	3	Verruciform	Not specified
(13)	Becheanu et al. [10]	Male/72	Lower	1	3	Yellowish elevated granular lesion	Atrophic gastritis
(14)	Arima [8]	Female/56	Lower	1	4	Yellowish elevated lesion	Biermer anemia, antral hyperplastic polyp with focal adenocarcinoma, atrophic gastritis
(15)	Min et al. [11]	Female/74	Middle	1	3	Verruciform	Atrophic gastritis, hyperlipidemia, dementia
(16)	Salamanca et al. [12]	Male/70	Upper	1	20	Verruciform	Hypertension, HCV, hemochromatosis, glottis cancer, hepatocellular carcinoma, tracheal cancer
(17)	Park et al. [13]	Male/67	Lower	1	2	White-yellowish elevated lesion	Ileocecal lymphoma
(18)	Bang et al. [14]	Male/70	Upper	1	3	Yellowish granular elevated lesion	Gastric and duodenal ulcer
(19)	Tsai et al. [15]	Male/62	Middle and lower	Multiple	2–10	Well-defined, fern-like, and yellowish lesions	Not specified
(20)	Díaz Del Arco et al. [16]	Female/56	Lower	1	13	Sessile polyp with white vascular surface	Segmental pneumonectomy for bronchiectasis, partial fundoplication for GERD
(21)	Our case	Female/60	Lower	1	1	Polyp/nodule	Diabetes, hypertension, hyperlipidemia, tubular colonic adenoma
